# Peritoneal opening during transanal endoscopic microsurgery: can preoperative positioning assessment improve intraoperative management?

**DOI:** 10.1007/s00464-026-12625-8

**Published:** 2026-02-04

**Authors:** Alberto Arezzo, Carlo Alberto Ammirati, Giovanni Distefano, Michele Barbiero, Simone Arolfo, Mario Morino

**Affiliations:** Department of Surgical Sciences, University of Turin, Corso Bramante 88, 10126 Turin, Italy

**Keywords:** Transanal endoscopic microsurgery (TEM), Patient positioning, Peritoneal opening, Peritoneal perforation, Outcomes, Predictors

## Abstract

**Background:**

Peritoneal opening (PO) during transanal endoscopic microsurgery (TEM) can cause the pneumorectum to collapse and complicate the procedure. As indications expand to larger, more proximal rectal lesions, understanding the real-world frequency, predictors, and consequences of PO is clinically important.

**Methods:**

We analysed a prospectively maintained single-centre database (January 1993–August 2025) of consecutive TEM/TEO resections. The primary exposure was PO; outcomes included abdominal conversion, complications, and length of stay (LOS). Multivariable logistic regression evaluated factors such as distal distance from the anal verge, maximal diameter, excision plane, and simplified lesion location (anterior, posterior, lateral, circumferential). The prone/supine position was examined overall and within the PO subgroup.

**Results:**

Of 1077 resections, PO occurred in 81/1,077 (7.5%). PO was more common with increasing distance from the anal verge: distal edge < 7 cm 2.3% (13/570) versus ≥ 7 cm 13.5% (68/502). Independent predictors included greater distance (OR 1.49 per cm; 95%CI 1.35–1.65; *p* < 0.001), larger diameter (OR 1.19 per cm; 95%CI 1.03–1.38; *p* = 0.022), full-thickness compared to submucosal excision (OR 4.03; 95%CI 1.39–11.66; p = 0.010), and circumferential versus anterior location (OR 4.78; 95%CI 1.61–14.19; p = 0.0049). Posterior location was protective (OR 0.26; 95%CI 0.13–0.53; *p* = 0.0002). Conversion occurred more frequently with PO (6.2%, 5/81) than without (0.1%, 1/992). LOS was longer with PO (median 5 [IQR 4–6] days) compared to without PO (median 4 [3–5] days; p < 0.001). The rate of complications did not differ significantly (any Dindo ≥ 1: 11.1% vs 7.3%; *p* = 0.19). Position was prone in 560/1,077 (52.2%) and supine in 513/1,077 (47.8%); PO was more common in prone (11.1%) than in supine (3.7%; p < 0.0001). Within the PO group, conversion was higher in the supine position (4/19, 21.1%) versus prone (1/62, 1.6%; *p* = 0.007), and laparoscopic-only conversions showed a borderline excess in the supine position (2/19 vs 0/62; *p* = 0.049).

**Conclusions:**

PO during TEM/TEO is infrequent, anatomically driven, and usually manageable with secure endoluminal repair. Careful surgical planning—considering distance, orientation, and size—and matching platform/position can reduce risk, with no evident increase in short-term morbidity.

**Supplementary Information:**

The online version contains supplementary material available at 10.1007/s00464-026-12625-8.

Transanal endoscopic microsurgery (TEM) and its rigid-platform successor, transanal endoscopic operation (TEO), offer magnified, stable visualisation and precise full-thickness local excision throughout the rectum, enabling organ-preserving surgery while avoiding the physiological burden of transabdominal resection. As indications have expanded to include larger and more proximal lesions, surgeons increasingly reach the peritoneal reflection, where full-thickness excision may open the peritoneal cavity (peritoneal opening, PO). This study provides a large, single-centre, rigid-platform (TEM/TEO) experience spanning three decades, validates a pragmatic 7 cm distance threshold with multivariable modelling that incorporates simplified lesion orientation, examines patient positioning (prone vs supine) as a correlate of PO and conversion, and proposes a graphical algorithm to standardise anticipation and escalation. PO has been considered an intraoperative setback because it can collapse the pneumorectum, complicate sutured closure, and raise theoretical risks of sepsis or tumour dissemination. However, the real clinical consequences—conversion, morbidity, and long-term cancer control—remain uncertain. Early multicentre experience suggested that, when recognised and closed primarily, peritoneal breach during TEM does not necessarily lead to significant short-term morbidity or compromised oncologic outcomes [1, 2]. Conversely, experience with transanal minimally invasive surgery (TAMIS) indicated caution in upper-rectal lesions: peritoneal entry was more frequent, platform choice was important, and some cases required platform change or abdominal assistance to achieve secure closure [3]. Albert and colleagues highlighted these practical challenges—loss of pneumorectum stability, less rigid instrumentation for intracorporeal suturing, and smoke/vision management—arguing that intraperitoneal closure is more manageable with rigid systems [4]. Issa and colleagues recommended selective adjunctive laparoscopy after PO—mainly to visualise and test the rectal repair—reporting low additional morbidity and the chance to repair occult leaks or place drains when necessary [5]. Similarly, Serra-Aracil and colleagues reported a 6–7% PO rate across a large transanal cohort and proposed a practical score to predict entry risk and guide closure strategies [6]. The development of minimally invasive endoluminal techniques has paralleled progress in endoscopic management of rectal neoplasia, including rectal neuroendocrine tumours, where comparative studies have refined indications and outcomes for organ-preserving resections [7].

Against this background, an updated evaluation based on a large, contemporary single-centre dataset can address three practical questions for surgeons who routinely perform TEM/TEO: (1) How often does PO occur today as case selection, technique, and technology have evolved? (2) Which patient, lesion, and technical factors independently predict PO in current practice? and (3) What are the measurable consequences of PO — conversion, length of stay, and complications graded by Dindo-Clavien — and what early oncologic signals are observed compared with cases without PO? Building directly on our 2013 experience [8], we now report an expanded cohort through 2025.

*Study aims*: Prespecified objectives were to: (1) quantify contemporary PO incidence; (2) identify independent predictors using multivariable modelling (distance, size, location, excision plane); (3) compare perioperative and early postoperative outcomes between PO and non-PO (NPO) cases; and (4) describe crude oncologic events within the PO subset to contextualise safety.

## Methods

We included all consecutive TEM or TEO resections (full-thickness or submucosal) for rectal lesions between January 1993 and August 2025 with PO status documented. Exclusions were: (i) procedures outside the rectum; (ii) non-excisional transanal procedures (e.g., biopsy, haemostasis only); (iii) cases performed with soft-port TAMIS; and (iv) missing PO status. Reporting conforms to STROBE guidelines. [9]

All resections were performed under general anaesthesia in a tertiary colorectal unit with an established transanal programme. Four colorectal consultants with dedicated TEM/TEO training performed or directly supervised every case; two senior surgeons led the programme throughout the study period, with fellows/residents executing parts of the procedure under direct supervision once competency was documented in simulation and cadaveric labs. A dedicated theatre team (anaesthesia, scrub, circulating nurse) routinely staffed these lists.

The primary exposure was peritoneal opening, defined as a documented intraoperative breach into the peritoneal cavity during local excision. Lesion location was abstracted from the operative record and categorised into simplified, mutually exclusive groups—anterior, posterior, right lateral, left lateral, or circumferential—to reflect the surgeon’s perspective with the patient in the operative position. Tumour geometry was recorded as the distance of the distal edge from the anal verge (centimetres) and the maximal diameter (centimetres), measured endoscopically or on the resected specimen when applicable. The excision plane was classified as full-thickness versus submucosal dissection based on operative intent and documented depth.

Process and clinical outcomes were predefined. Conversion was defined as escalation to abdominal access (open or laparoscopic) for any reason related to the initial local excision; changes limited to the transanal platform (e.g., switch to TEO) were not considered conversions. Postoperative outcomes included the Dindo–Clavien grade of the most severe in-hospital event, targeted complications of interest (bleeding requiring transfusion or reoperation, urinary retention, bladder lesion, anastomotic or rectal leak, rectovesical fistula), creation of a stoma, and length of stay from the operation to discharge (days). Oncologic events were documented as crude (unadjusted) local and distant recurrences as recorded in follow-up; the database does not consistently specify the anatomical site of distant failure.

Drawing on anatomical considerations and prior literature, we predefine the following hypotheses:

1. The risk of peritoneal opening increases progressively with greater distal distance from the anal verge and the anterior or circumferential orientation.
2. Larger lesions and full-thickness excision raise the chance of peritoneal opening.
3. When the peritoneal opening is recognised and securely repaired, postoperative morbidity remains comparable to cases without peritoneal entry, with a modest increase in operative complexity and length of stay.

Continuous variables are summarised as medians with interquartile ranges and compared between groups using non-parametric tests. Categorical variables are presented as counts and percentages and compared with χ^2^ or Fisher’s exact tests, as appropriate. To identify independent predictors of PO, we fitted a multivariable logistic regression including the a priori covariates of distal distance, maximal diameter, and excision plane. We simplified lesion location by categorising anterior as the reference category. Adjusted effects are reported as odds ratios (ORs) with 95% confidence intervals. Missing data were handled with complete-case analysis for the regression model; descriptive denominators vary according to available data and are stated in the tables. Because PO-related conversions appeared concentrated early in institutional experience, we performed a learning-curve sensitivity analysis that contrasted the distribution of conversions in the first 100 chronological cases with the remainder of the series. All tests were two-sided, with *p* < 0.05 considered statistically significant. Analyses were performed on a locked data export dated August 2025.

Under the institutional policy for minimal-risk retrospective studies using fully de-identified data, formal IRB/ethics review is not required. The project was conducted as part of our unit’s service evaluation/quality-improvement activity. No direct identifiers were retained; data were pseudonymised prior to analysis and handled in accordance with GDPR and the Declaration of Helsinki. The requirement for individual consent was therefore waived.

## Results

### Cohort and baseline characteristics

Of 1077 resections. PO occurred in 81 cases (7.5%). Baseline features by PO status are summarised in Table 1. Groups were balanced for sex and age, whereas lesions with PO were located more proximally (median distal edge 9 cm [IQR 7–11] vs 6 cm [5–8]; *p* < 0.001), required longer operative time (90 [60–130] vs 60 [40–90] minutes; p < 0.001), and yielded a longer length of stay (5 [4–6] vs 4 [3–5] days; p < 0.001). The proportion of full-thickness excisions was high in both groups (94% vs 90%; *p* = 0.26).

**Table 1:** Patient characteristics and perioperative data (PO vs NPO)

Variable	PO (*n* = 81)	NPO (*n* = 996)	p
Male sex, n/N (%)	44/81 (54.3)	578/996 (58.3)	0.485
Age, years, median (IQR)	71 (62–77)	69 (60–77)	0.265
Distal distance, cm, median (IQR)	9 (7–11)	6 (5–8)	< 0.0001
Diameter, cm, median (IQR)	3 (2–4)	3 (2–4)	0.490
Operative time, min, median (IQR)	90 (60–130)	60 (40–90)	< 0.0001
Full‑thickness excision, n/N (%)	76/81 (93.8)	895/996 (90.2)	0.428
Conversion to abdominal surgery, n/N (%)	5/81 (6.2)	1/996 (0.1)	< 0.0001
Length of stay, days, median (IQR)	5 (4–6)	4 (3–5)	< 0.0001

Baseline features comparing peritoneal opening (PO) with no peritoneal opening (NPO)

### Risk as a function of distance

**Fig. 1:**
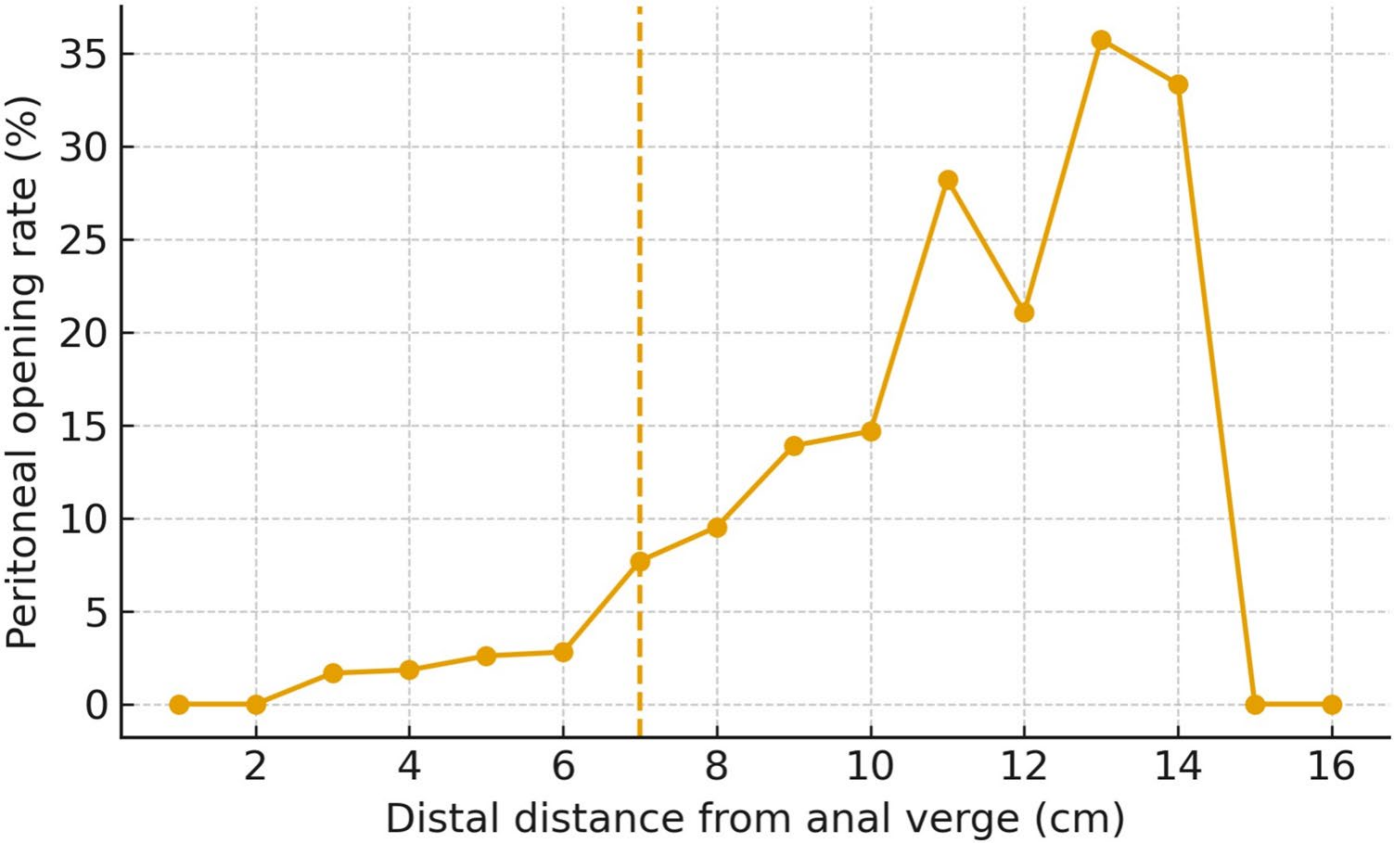
Risk of peritoneal opening by the distal distance from the anal verge

The empirical probability of PO increased monotonically with distal distance from the anal verge (Fig. 1). A pragmatic cut-off separated risk strata: PO was 2.3% when the distal edge was < 7 cm (13/572) versus 13.5% when ≥ 7 cm (68/504) (Table 2).

**Table 2:** Postoperative morbidity by Dindo–Clavien

Grade	PO (*n* = 81)	NPO (*n* = 996)	p
0	72	922	0.194
1	1	6	0.424
2	0	28	0.263
3a	2	15	0.373
3b	6	23	0.018
Any complication (≥ 1)	9	72	0.194

Distribution of complications by grade with per‑grade two‑group tests and overall distribution p-value (χ^2^) = 0.034

### Predictors of peritoneal opening

**Fig. 2:**
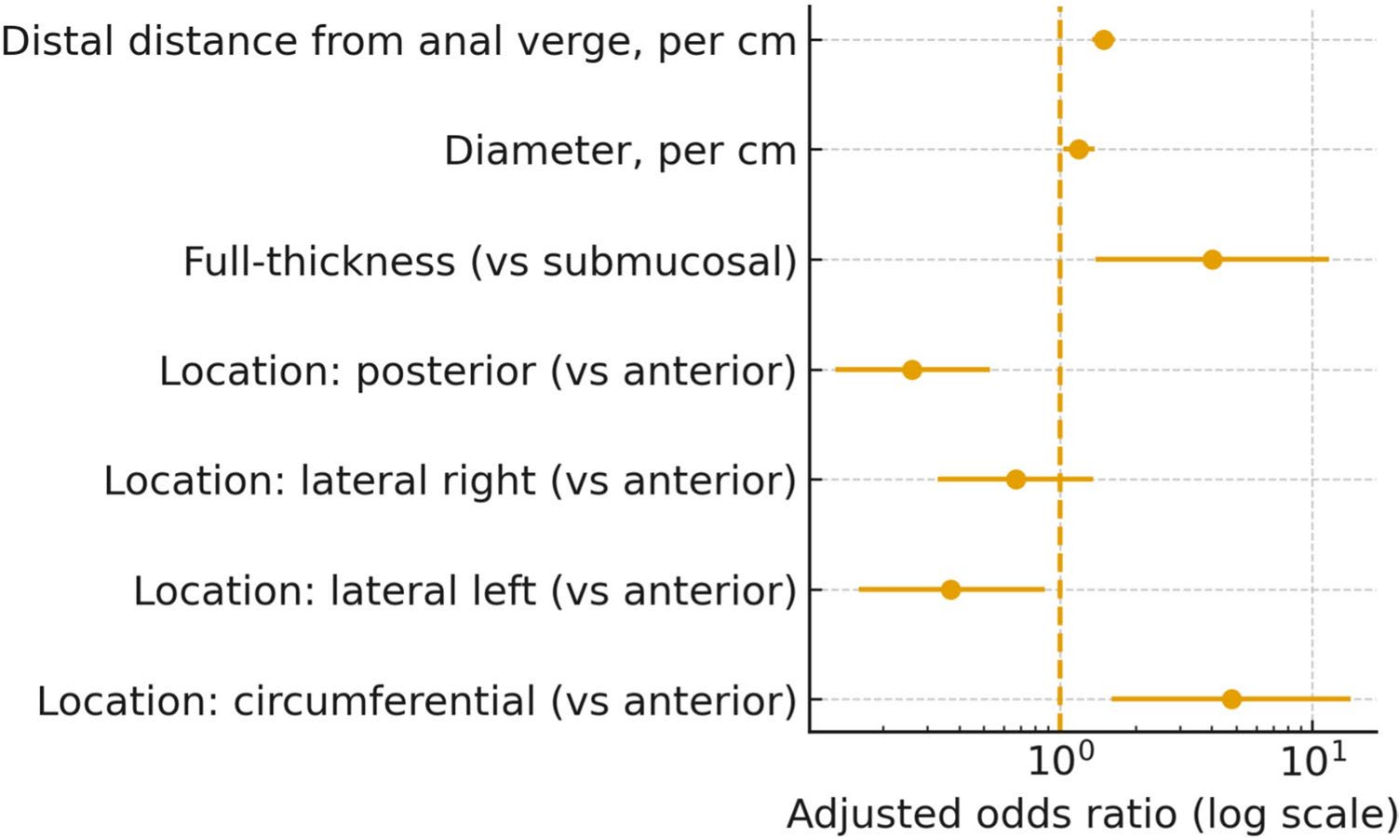
Predictors of peritoneal opening

In multivariable logistic regression, independent predictors of PO were greater distal distance (OR 1.49 per cm; 95%CI 1.35–1.65; *p* < 0.001), larger diameter (OR 1.19 per cm; 1.03–1.38; *p* = 0.022), full-thickness excision versus submucosal (OR 4.03; 1.39–11.66; *p* = 0.010), and circumferential location versus anterior (OR 4.78; 1.61–14.19; *p* = 0.0049). Posterior location was protective (OR 0.26; 0.13–0.53; p = 0.0002). Adjusted effects are detailed in Table 3 and visualised in Fig. 2.

**Table 3:** Predictors of peritoneal opening (multivariable logistic regression)

Predictor	Adjusted OR (95% CI)	p
Distal distance from anal verge, per cm	1.49 (1.35–1.65)	< 0.0001
Diameter, per cm	1.19 (1.03–1.38)	0.022
Full‑thickness (vs submucosal)	4.03 (1.39–11.66)	0.010
Location: posterior (vs anterior)	0.26 (0.13–0.53)	< 0.0001
Location: lateral right (vs anterior)	0.67 (0.33–1.36)	0.269
Location: lateral left (vs anterior)	0.37 (0.16–0.87)	0.023
Location: circumferential (vs anterior)	4.78 (1.61–14.19)	0.005

Adjusted odds ratios from a logistic model including distal distance, diameter, excision plane, and lesion location (anterior baseline). Excision reported as full‑thickness vs submucosal (inverted from model coding)

### Patient positioning

Position was recorded as supine in 515/1,077 (47.8%) and prone in 562/1,077 (52.2%). PO occurred more often during prone cases (62/562, 11.1%) than supine (19/515, 3.7%), Fisher’s exact *p* < 0.0001; odds ratio 3.24. Within groups, prone positioning was used in 62/81 (76.5%) PO cases versus 498/996 (50.2%) NPO cases.

### Intraoperative management and conversion

Abdominal conversion (open or laparoscopic) was more frequent with PO (6.2%, 5/81) than without (0.1%, 1/996), consistent with greater technical demand at the peritoneal reflection (Table 1). Conversions clustered early in institutional experience: among PO cases, 3/7 in the first 100 chronological operations converted versus 2/74 thereafter (learning-curve sensitivity).

### Era, platform and operator-stratification

Peritoneal opening increased over calendar eras, from 4.2% in 1993–2004 to 9.1% in 2015–2025 (*χ*^*2*^ = 9.8, *p* = 0.0078), consistent with the progressive inclusion of larger and more proximal lesions (Table S2A). PO was more frequent with TEO than with TEM (9.1% vs 4.2%; χ^2^ = 8.3, *p* = 0.039), whereas major complication rates remained similar across platforms (Table S2B). No significant differences in PO rates were observed between surgeon groups (*p* = 0.60), indicating that PO was primarily driven by case-mix and anatomy rather than by operator (Table S2C).

### Position vs conversion/laparoscopy

**Fig. 3:**
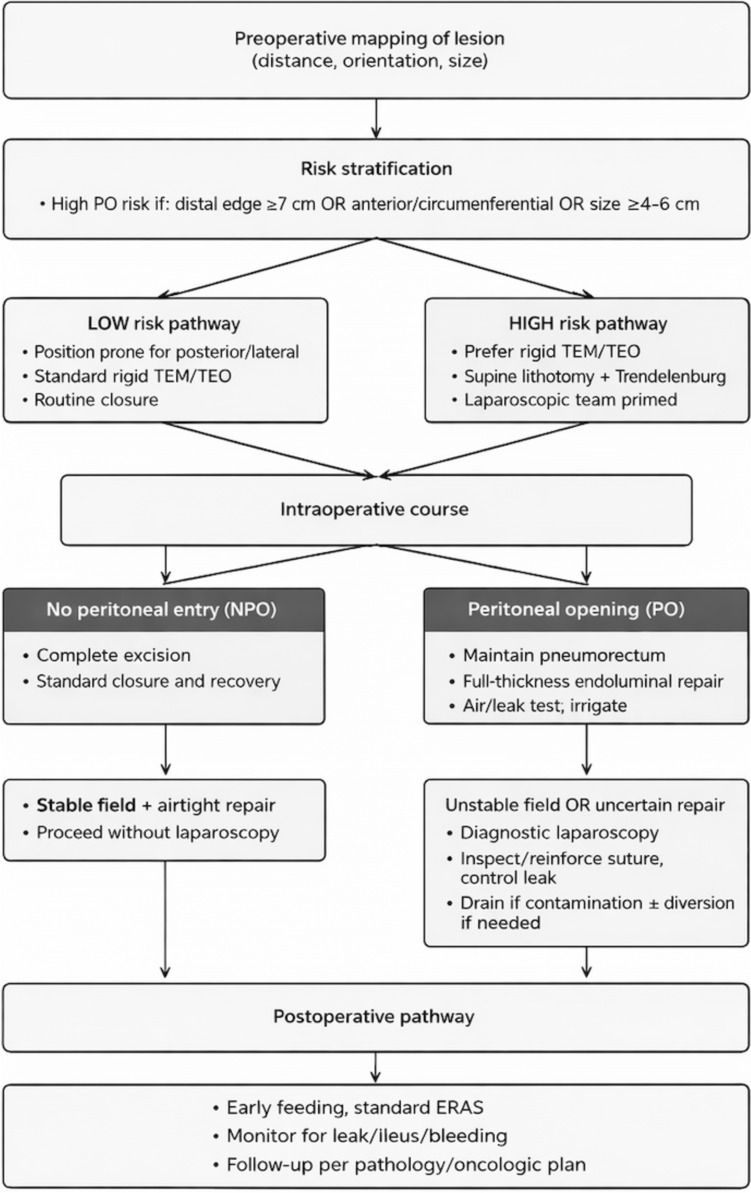
Risk-stratified algorithm for anticipation and management of peritoneal opening during TEM/TEO. Anticipate–Close–Escalate algorithm for peritoneal opening (PO). Risk triage by distance–orientation–size with a pragmatic ≥ 7 cm distal cutoff. Prefer rigid platforms near/above the peritoneal reflection; tailor patient positioning to lesion orientation; and adopt low-friction diagnostic laparoscopy to inspect, reinforce and irrigate when endoluminal repair is uncertain or the field is unstable

Overall, abdominal conversion (either open or laparoscopic) was rare and did not significantly differ by position: supine 4/515 (0.8%) vs prone 2/562 (0.4%); Fisher’s exact p = 0.434 (OR 2.19). Laparoscopic conversion was similarly uncommon and evenly distributed: supine 1/515 (0.2%) vs prone 1/562 (0.2%); *p* = 1.000 (OR 1.09). In the PO subset, conversion was numerically higher in the supine position (4/19, 21.2%) compared to prone (1/62, 1.6%), with statistical significance; *p* < 0.0001 (OR 16.27, 95% CI 1.69–156.35). Laparoscopic conversion within PO was 2/19 (10.6%) for supine versus 0/62 (1.6%) for prone; *p* = 0.049. To facilitate adoption, we provide a stepwise algorithm (Fig. 3) that links preoperative risk stratification (distance–orientation–size) to platform/position selection and a standardised escalation pathway (diagnostic laparoscopy when the endoluminal repair is uncertain or the field is unstable).

### Postoperative outcomes

The distribution of Dindo–Clavien grades showed no significant difference between groups (Table 2). Any complication (grade ≥ 1) was observed in 11.1% (9/81) of the PO group and 7.3% (72/996) of the non-PO group; the difference was not statistically significant (Fisher’s exact *p* = 0.19). Specific events in the PO group included leaks (2.5%), rectovesical fistula (1.2%), and stoma creation (2.5%). Length of stay remained significantly longer following PO, as noted above (Table 1).

### Oncologic signals within the PO subset

Crude recurrence within PO is shown in Table 4. Among invasive adenocarcinomas with recorded follow-up (*n* = 22), local recurrence occurred in 36.4% (8/22) and distant recurrence in 18.2% (4/22). Benign lesions showed 7.7% (4/52) local recurrence and 0% (0/52) distant recurrence; carcinoma in situ (Tis) and other malignancies had no recorded events within the available follow-up.

**Table 4:** Crude oncologic outcomes by histology class in peritoneal opening (PO) vs non-PO groups (locked export August 2025)

Histology class	PO n	PO local, n (%)	PO distant, n (%)	Non-PO n	Non-PO local, n (%)	Non-PO distant, n (%)
Invasive adenocarcinoma	22	8 (36.4)	4 (18.2)	263	45 (17.1)	29 (11.0)
Carcinoma in situ (Tis)	2	0 (0.0)	0 (0.0)	54	3 (5.6)	0 (0.0)
Benign	52	4 (7.7)	0 (0.0)	636	33 (5.2)	0 (0.0)
Other malignancies	5	0 (0.0)	0 (0.0)	51	2 (3.9)	1 (2.0)
Overall	81	12 (14.8)	4 (4.9)	992	83 (8.4)	30 (3.0)

*PO* peritoneal opening

Events are crude (unadjusted). Denominators reflect cases with recorded follow-up for recurrence variables

### Oncologic outcome comparator

When compared with cases without peritoneal opening, crude oncologic event rates were numerically higher in PO for invasive adenocarcinoma, but differences were not observed for carcinoma in situ or benign lesions. Among invasive cancers, local recurrence occurred in 36.4% (8/22) of PO cases versus 17.1% (45/263) in non-PO, and distant recurrence in 18.2% (4/22) versus 11.0% (29/263), respectively (Table 4). In contrast, recurrence rates for Tis and benign lesions were low and similar between PO and non-PO groups, with no distant failures recorded for non-invasive disease.

## Discussion

This updated single-centre cohort offers a long-term, current perspective on peritoneal opening (PO) during transanal endoscopic local excision. Several messages are consistent and directly applicable to clinical practice. First, PO remained uncommon overall (7.5%), with the risk mainly influenced by anatomy and lesion geometry rather than patient factors. Second, when PO occurred and was securely closed, severe morbidity was not significantly increased. However, there was a modest impact on length of stay and a small but real risk of conversion persisted. Third, the risk increases progressively with distance from the anal verge, with a simple 7 cm threshold, and the final multivariable model confirms that distance, lesion size, excision plane, and circumferential orientation are the key, independent predictors. These findings endorse a pragmatic approach based on anticipation, meticulous closure, and selective escalation. PO-risk is primarily anatomical and lesion-related, with outcomes relying more on recognition and reliable closure than merely on the occurrence of peritoneal opening. They also suggest a significant platform signal: rigid TEM/TEO maintains a sealed working space, straightforward instrumentation, and consistent suturing ergonomics at the peritoneal reflection, whereas TAMIS—despite its versatility—may encounter greater instability of pneumorectum and more challenging full-thickness closure within the peritoneal cavity, prompting a lower threshold for adjunctive laparoscopy or conversion in complex upper-rectal cases [4–6]. When these principles are integrated into organ-preserving pathways, recent series indicate they can be delivered with acceptable oncologic safety in carefully selected early rectal cancers [10, 11].

Out of 1,077 procedures with recorded PO status, the overall PO rate was 7.5%, showing clear differences by site: 10.4% for anterior lesions, 9.8% for right lateral, 5.1% for left lateral, 3.0% for posterior, and 28.0% for circumferential involvement. This pattern matches surgical anatomy: posterior lesions usually stay extraperitoneal; anterior and circumferential targets approach or cross the peritoneal reflection earlier; and laterally oriented dissections can shift anteriorly as the dissection progresses. The analysis confirms this understanding. For every centimetre the distal edge lies more proximally, the adjusted odds of PO increase by about 50%; each additional centimetre of diameter results in a smaller but meaningful rise in risk; full-thickness excision increases risk fourfold compared to submucosal dissection; and circumferential lesions remain high-risk even after accounting for distance and size. Posterior orientation offers protection. Notably, sex and age did not distinguish the groups in our dataset (Table 1), suggesting that preoperative counselling and operative planning should mainly focus on lesion-specific factors. Our findings align with earlier TEM experience in large institutional series and technical developments that define the capabilities and limits of local excision [12, 13].

Clinically, PO was associated with longer operative times and an increase of one day in median length of stay (5 vs 4 days). Abdominal conversion was rare overall but significantly more common in the PO group (6.2% vs 0.1%) and tended to occur early during institutional experience. Complications graded by Dindo–Clavien were similar between groups, and specific adverse events in PO — leak, rectovesical fistula, stoma — were uncommon in absolute terms (Table 2). These observations support a practical message: when PO is recognised and closed skillfully, it is usually a manageable event with the main costs being extra time and a slightly longer admission, rather than a major increase in morbidity. This approach fits within the broader context of oncologic and organ-preservation strategies, where selection, response assessment, and follow-up play significant roles in outcomes, at least as much as the intraoperative course [14–17]. Consistent with this, our recent single-centre analyses reported positive safety and structured oncologic results after completion TEM for incidental pT1 disease and after transanal local excision in selected pT2N0 patients, highlighting the importance of careful selection and monitoring [10, 11].

The method of access influences both the chance of peritoneal entry and the dependability of closure after entry. Our programme mainly utilises a rigid-platform approach (TEM/TEO). Rigid platforms stabilise the pneumorectum, standardise suturing ergonomics, and maintain optical discipline at the peritoneal reflection; these features are key to achieving secure intraperitoneal closure. TAMIS, although flexible for low/mid-rectal procedures, can encounter field instability and pose more challenging suturing mechanics near the reflection. Therefore, TAMIS requires a lower threshold for adjunctive laparoscopy or conversion when exposure is limited, aligning with cautious and pragmatic views on peritoneal entry during transanal endoscopic resection [3–5, 18, 19].

Positioning should be used proactively and tailored to lesion orientation and height. The lithotomy position usually optimises exposure for posterior or posterolateral lesions (often extraperitoneal), whereas the prone jack-knife position facilitates access to anterior or higher targets where reflection is encountered sooner. Peritoneal opening was significantly associated with the prone position, which is explained by the fact that we adopt this position for anterior and proximal lesions, inherently carrying a higher risk of peritoneal involvement. In our experience, abdominal wall pressure against the rectum in the prone position may help maintain pneumorectum stability, making secure closure easier if a peritoneal breach occurs. In our data, overall conversion was rare and not dependent on position. However, within PO, there was a clear association between the supine position and conversion, with a borderline excess of laparoscopic conversion—biologically plausible signals given case selection (anterior/high targets) and limited by small denominators. In summary, we identify a statistically significant link between supine position and conversion among PO cases, with a borderline excess of laparoscopic conversions influenced by case selection and small sample sizes. These observations are synthesised into a pragmatic, risk-stratified management pathway that links lesion anatomy to positioning, platform choice and escalation strategy (Fig. 3). These observations support practical guidelines and technical notes on managing peritoneal entry during TAMIS and related approaches [1, 3–5, 13, 18, 19]. Our analysis also encourages a deeper examination of the relationship between patient position, conversion, and peritoneal opening, suggesting that patient positioning is influenced more by tumour characteristics and location than the device used for transanal dissection (TEO vs. TAMIS). This challenges the common perception that one disadvantage of TEM is the need for an non-ergonomic patient position.

Within the PO subset, crude recurrence rates were higher for invasive cancers than for benign lesions. However, these figures are not adjusted for tumour or treatment covariates and lack site-specific adjudication of distant failure. Without site data, distant events cannot be attributed to peritoneal dissemination. The most methodologically robust interpretation is therefore one of evidence-based neutrality: secure repair of PO does not appear to increase short-term morbidity; oncological outcomes are likely influenced by tumour biology and resection quality. This perspective aligns with historical observations following local excision and with modern organ-preservation frameworks (watch-and-wait, local excision after neoadjuvant therapy), which rely on comprehensive selection, response assessment, and standardised reporting [14–17]. Sentinel-node concepts and fluorescence-guided nodal mapping/harvesting demonstrate evolving methods to characterise biology and customise treatment when organ-preservation is pursued [20, 21]. Contemporary surveys and trials also demonstrate how practice patterns and supportive technologies are transforming the landscape of local excision and surveillance [22–24]. Similar selection criteria are evident in rectal neuroendocrine tumours, where comparative endoscopic strategies refine size- and site-based indications for organ-preserving treatment [7].

Beyond platform and position, innovations such as transanal endoscopic submucosal dissection and robotic assistance expand the technical boundaries for en bloc and full-thickness resections. However, they also highlight the ergonomic and insufflation challenges of secure intraperitoneal suturing near the reflection [22, 23]. Similar experience from endoscopic full-thickness management of gastric stromal tumours emphasises the same principle that platform stability and reliable defect closure determine safety once the serosa is traversed—an insight applicable to intraperitoneal TEM/TEO work [25].

Conversions in PO clustered early and then became infrequent—aligning with a systems-level learning effect. Structured simulation (including induced field instability), deliberate practice with steep-angle endoluminal suturing, and standardised “if we enter the peritoneum” briefs customised for platform/position appear to be key factors. These programme features probably support the sustained decline in conversion and reflect broader lessons from advanced transanal and laparoscopic colorectal surgery [12, 18, 19].

## Limitations

This is a single-centre experience based on rigid-platform work; caution is advised when extrapolating to TAMIS-dominant settings [3–5, 18–20, 26, 27]. The analysis is retrospective (despite prospective data collection), complete-case modelling might bias estimates if missingness is not random, and conversion definitions excluded platform swaps without abdominal access. Oncologic endpoints are crude and lack site adjudication; they generate hypotheses rather than causal claims. These limitations indicate the need for targeted, prospective studies with harmonised definitions and detailed follow-up.

Strengths include the size and temporal breadth of a single-technology programme; prospective, granular capture of exposure and operative details; continuity with an earlier series enabling temporal benchmarking; and the practical translation of findings into a usable algorithm.

Next steps include a prospective, multi-centre registry with harmonised definitions (including site-adjudicated distant failure), era/platform stratification, and standardised escalation triggers; external validation of the risk model and the 7 cm rule; and pragmatic evaluation of adjunctive laparoscopy policies. In our programme, we have adopted pre-list huddles with an explicit ‘PO-risk’ plan (platform/position, leak test, laparoscopy-on-standby) and targeted simulation for steep-angle endoluminal suturing near the reflection.

In conclusion, peritoneal opening during transanal endoscopic local excision should be viewed as a predictable, anatomy-related event rather than an inherent failure. Surgeons should anticipate risk by considering the distance–orientation–size triad: lesions with a distal edge ≥ 7 cm and anterior or circumferential orientation require a deliberate “PO-risk” plan that emphasises a rigid-platform (TEM/TEO) alongside a prepared laparoscopic team. The platform and patient positioning should be matched to the lesion; rigid systems with customised positioning offer the most reliable conditions for secure intraperitoneal repair. TAMIS near the peritoneal reflection benefits from an anticipatory approach and a lower threshold for adjunctive laparoscopy [3–5, 19, 20, 26, 27]. Escalation should be standardised: when the field becomes unstable or the repair uncertain, diagnostic laparoscopy to inspect, reinforce, and irrigate should be a straightforward step. Using this approach, teams can expect modest costs—slightly increased operative time and approximately + 1 day in-hospital—without introducing new hazards, as long as the defect is recognised and securely closed [1, 2, 6, 8, 9, 12]. Peritoneal opening during transanal endoscopic local excision is an anatomy-predicted, manageable event; anticipating risk (distance–orientation–size), matching platform and position to the lesion, and standardising low-threshold adjunctive laparoscopy contain its impact to time and a modest increase in length of stay.

## Supplementary Information

Below is the link to the electronic supplementary material.

Supplementary file1 (DOCX 37 KB)
