# Supplementary material for: Peritoneal opening during transanal endoscopic microsurgery: can preoperative positioning assessment improve intraoperative management?
Source: Surg Endosc. 2026 Feb 4;40(4):3263–72. doi: 10.1007/s00464-026-12625-8 (PMC13053563; doi:10.1007/s00464-026-12625-8)
Supplement: Supplementary file 1 — Supplementary file1 (DOCX 37 KB) [file 464_2026_12625_MOESM1_ESM.docx]

**Supplementary Table S2. Era, platform, and operator stratification for peritoneal opening (PO) and key outcomes (locked export August 2025).**

A) By calendar era

| Era | n | PO, n (%) | Conversions, n (%) | Median operative time (min) | Median LOS (days) | Major complications (Clavien ≥III), n (%) |
| --- | --- | --- | --- | --- | --- | --- |
| 1993–2004 | 382 | 16 (4.2) | 5 (1.3) | 60 | 4 | 16 (4.2) |
| 2005–2014 | 416 | 30 (7.2) | 3 (0.7) | 60 | 3 | 18 (4.3) |
| 2015–2025 | 279 | 25 (9.1) | 5 (1.8) | 60 | 3 | 12 (4.3) |
| Across eras: χ² for PO = 9.8, p = 0.0078 |  |  |  |  |  |  |

B) By platform

| Platform | n | PO, n (%) | Conversions, n (%) | Major complications (Clavien ≥III), n (%) |
| --- | --- | --- | --- | --- |
| TEM | 310 | 13 (4.2) | 3 (1.0) | 14 (4.5) |
| TEO | 738 | 67 (9.1) | 3 (0.4) | 31 (4.2) |
| Flexible transanal platform | 26 | 1 (3.8) | 5 (19.2) | 1 (3.8) |
| DaVinci SP assistance | 3 | 0 (0.0) | 1 (33.3) | 0 (0.0) |
| Across platforms: χ² for PO = 8.3, p = 0.039 |  |  |  |  |

*Note: Small numbers in flexible/DaVinci SP rows; interpret conversion rates with caution.*

C) By operator group

| Operator group | n | PO, n (%) |
| --- | --- | --- |
| Morino | 423 | 30 (7.1) |
| Arezzo | 483 | 37 (7.7) |
| Others | 171 | 11 (6.4) |
| Across groups: χ² = 1.0, p = 0.60 |  |  |

Abbreviations: PO, peritoneal opening; LOS, length of stay; TEM, transanal endoscopic microsurgery; TEO, transanal endoscopic operation.
